# Nurse workforce change and metropolitan medically underserved areas in the United States

**DOI:** 10.1186/s12913-025-12228-4

**Published:** 2025-01-15

**Authors:** Diana Bowser, Kaili Mauricio, Brielle Ruscitti

**Affiliations:** William F. Connell School of Nursing, Boston College, 140 Commonwealth Ave, Chestnut Hill, MA 02467 USA

**Keywords:** Nurse workforce, Medically underserved areas, Workforce retention, Nurse mobility, Nurse occupation growth

## Abstract

**Background:**

The continued healthcare crisis in the United States (US) is worrisome, especially as workforce shortages, particularly for nurses, are highlighted, often in some of the highest need areas. As the need for healthcare services grows, especially for services that nurses can deliver, the inability to meet those needs exacerbates existing disparities in access to care and can jeopardize the quality and timeliness of healthcare delivery in underserved communities. Prior investigations have used varying definitions to describe underserved, under-resourced, rural, or health professional shortage areas to examine the relationship between these areas and workforce shortages. Therefore, this study examines the relationship between changes in the nursing labor force changes and metropolitan medically underserved areas (MUA), defined by Health Resources and Services Administration (HRSA).

**Methods:**

Secondary data were utilized to conduct descriptive and regression analyses of the nursing workforce population in metropolitan statistical areas from 2012 to 2022. The key outcome variable for the analyses was nurse workforce change per 10,000 population. Occupational Employment and Wage dataset from the Bureau of Labor Statistics was used to determine the number of nurses employed, at the level of the metropolitan statistical area from 2012 to 2022. The Index of Medical Underservice was extracted for each MUA from HRSA and geographically weighted to the metropolitan area.

**Results:**

The results of descriptive trends for nursing professions show that all nursing occupations reviewed have experienced positive change over both five- and ten-year periods. However, the results of nurse change models show that neither the change in Registered Nurses nor Nurse Practitioners is correlated with medically underserved areas.

**Conclusions:**

These results emphasize the need for adaptive strategies in the nursing workforce to respond to the evolution of healthcare requirements over time. The findings from this study suggest the need for careful planning in workforce policy and education to grow the nurse workforce needs to meet evolving healthcare needs effectively.

**Supplementary Information:**

The online version contains supplementary material available at 10.1186/s12913-025-12228-4.

## Introduction

Access to healthcare and healthcare infrastructure varies widely across the United States (US), illustrated by a range of inequities in the US healthcare system, including but not limited to nursing workforce shortages. While nurses became the nation’s “healthcare heroes” during the COVID-19 pandemic, a number of factors, including COVID-19 exposure, personal protective equipment availability, fear, depleted resources, and family and personal reasons, caused many nurses to leave the profession [1–4]. Nursing homes and other long-term care facilities were especially impacted by the nursing shortage during the COVID-19 pandemic [5]. While the COVID-19 pandemic highlighted the nurse shortage, trends in nursing turnover had begun to increase prior to the pandemic, due to demographic factors, insufficient staffing, a lack of experience, lack of nursing schools in high-need areas, low salaries, and low job satisfaction [6–8]. Many of these factors were exacerbated in areas that were already high-need and medically underserved.

Medically underserved areas (MUA), defined by the Health Resources and Services Administration (HRSA), are identified as geographic areas and populations with a lack of access to primary care services [9]. MUAs included in this analysis are shown in Annex A1. MUAs are identified either through predetermined criteria (density of primary care physicians per 1,000 people, proportion of residents living below the federal poverty level, percentage of the population aged 65-years and older, and the area's infant mortality rate) or via a discretionary process based on unique circumstances or considerations [9]. MUAs tend to be located in areas with a high number of patients with more unique or complex medical needs and delayed or forgone care, resulting in barriers in access to care for many patients [10–12]. While research shows that many of the healthcare needs in MUAs can be addressed through primary care services [13], which can be increasingly delivered by nurses [14, 15], to the authors knowledge, there have been no studies examining the number of nurses working in and moving to MUAs, as defined by the HRSA.

The continued healthcare crisis in the US is worrisome, especially as workforce shortages, particularly for nurses, are highlighted, often in some of the highest-need areas. While some studies have shown a rebound in nursing workforce following the COVID-19 pandemic in 2022 and 2023 [16], the US Department of Health and Human Services has projected seven states will have a shortage of RNs in 2030, with four of these states having a deficit of 10,000 nurses or more [17]. Georgia and Minnesota report the highest rates of shortages, with both states having nursing shortages of over 25% [5]. Furthermore, as of 2020, the South, as defined by the US census, had the lowest per-population availability in the nation for certified nurse-midwives, and the highest maternal mortality [18]. Areas with nursing shortages tend also to have similar patterns with other health related indicators like infant mortality. Alabama, with one of the highest infant mortality rates in the US, faces severe nurse and physician shortages, resulting in a 9% loss of labor and delivery services from 2004 to 2014 [19, 20]. Additionally, research on health professional shortage areas (HPSAs) in the Mississippi delta region, which contains 14% of all HPSAs in the US, finds that nearly 16,000 additional primary care providers would be needed to overcome workforce shortages, and despite this, the number of NPs working in this region has remained stable over a decade [21]. Studies have also examined the link between HPSAs and nurse work force trends related to burnout and job satisfaction, finding mixed results, emphasizing the need to understand the workforce overall [22–24]. While there has been some federal and state level initiatives to incentivize recently graduated nurses to move to underserved areas, the impacts of such programs for RNs is unknown and requires further research [25].

While this study examines nurse shortages in the US, similar challenges are present across the globe. The International Council of Nurses has noted there is currently a global shortage, and the shortage is not expected to shrink by 2030 [26]. The healthcare worker shortage extends beyond nurses, with the World Health Organization noting both the unequal distribution and general shortage of healthcare workers globally [27, 28]. This inequitable distribution at the international level plays out in the US, where many areas have a shortage of primary healthcare services, yet very few have a surplus [29].

While many studies have examined trends in nursing shortages or broad definitions of underserved and shortage areas [21, 30, 31], this paper aims to examine trends in nursing workforce in comparison to MUAs as defined by HRSA. Given the high need for healthcare services in MUAs, many of which can be delivered by nurses, this study aims to map changes in the nursing labor force and change at the MUA level.

## Methods

The Occupational Employment and Wage (OEW) dataset from the Bureau of Labor Statistics (BLS) [32], a comprehensive dataset of employment and wages in occupational categories and geographic areas, was used to extract data on the number of nurses employed for the following categories: registered nurses (RN), nurse practitioners (NP), nurse midwives, and nurse anesthetists. While the BLS included data on additional nurse roles including licensed practical nurses, certified nursing assistants, and nursing instructors, this analysis was limited to roles listed above due to workforce trends and data availability. Data were extracted at the level of the metropolitan statistical area (MSA) over the period 2012–2022 to create an estimate of the number of workers within the nurse categories referenced above for each MSA for each year. The MSA-level data from the BLS were then combined with population data from the Census Bureau American Community Survey for the appropriate years. In the US, MSAs were defined by the U.S. Office of Management and Budget (OMB) and used by the (BLS) as an area that contains at least one urbanized area with a population of 50,000 or more. Additionally, MSAs may have included adjacent communities that have a high degree of social and economic integration with the core urban area [33].

As previously outlined, MUAs were designated in two ways, one being predetermined criteria measured across four dimensions: the density of primary care physicians per 1,000 people, the proportion of residents living below the federal poverty level, the percentage of the population aged 65 years and older, and the area's Infant Mortality Rate. These criteria then contribute to the calculation of the MUA score, which if below a specified threshold, designated the area as an MUA. MUAs were also designated via a discretionary process, where state or federal authorities grant special exceptions based on unique circumstances or considerations. MUA designations are reviewed and confirmed every three years, with the most recent data in this analysis from 2022. MUA scoring ranged from zero to 100 with lower scores translating to areas demonstrating a more medically underserved status. MUA scores were extracted from the HRSA and calculated for each geographic area used throughout this analysis.

Since the MSAs utilized by the BLS had different geographic boundaries than the MUAs, MUA scores for MSAs were calculated using a weighted average MUA score for each MSA. An area weighting was utilized, which captured the percent of the total geographic area of each MUA within the larger MSA to create a weighted MUA score for each MSA. As part of the weighting, all geographic areas within MSAs that were not given an MUA score by HRSA were assigned the highest score (100) to capture areas with MSAs that were not medically underserved. Geographic research has noted the challenges in transforming the scale of spatial data to comparable levels [34, 35], and while the recommendation for spatial weighting is ideal and population-based, the lack of population at the MUA level precluded any population-weighted scores at the MSA level. This approach assumed relatively uniform population density across MSAs where the MUAs were located. Nonmetropolitan areas were not included in the analysis, excluding approximately 12% of RNs and 14% of NPs that worked in rural areas in 2022. This study focused on metropolitan areas due to the following data limitations within nonmetropolitan areas. The BLS OEW dataset had limited data on the number of nurses in varying professions in nonmetropolitan areas. Nurse growth in the limited nonmetropolitan areas of a state may not align with the areas of need as defined by the respective MUAs. Furthermore, the MUAs often were limited to small areas within the nonmetropolitan areas, so the geographic weighting strategies described above would result in non-comparable MUA scores across nonmetropolitan areas. The metropolitan-only analysis included 72% of all 2022 designated MUAs and while there was a rural shortage of healthcare workers [36], this analysis focused on the relationship between nurse growth and need in metropolitan areas. All 393 metropolitan statistical areas had data at the MUA level, and 376 out of the 393 had data on nurse growth from the BLS for the years analyzed. Annex A2 depicts the geographical distribution of weighted MUA scores by quintile brackets, with quintile one represented by a score of 43.8–62.0 (highest medically underserved areas) and quintile five represented by a score range of 94.3–100.0 (lowest medically underserved areas).

Using the OEW data on the MSA nurse workforce over the period 2012–2022, 5-year (2017–2022) and 10-year (2012–2022) nurse change rates (total and per 10,000 residents) were calculated for the following nurse categories: RNs, NPs, and two additional nurse categories: nurse anesthetists and nurse midwives. Due to the limited number of MSAs with a substantial presence of nurse anesthetists or nurse midwives, a regression analysis was not feasible, therefore, descriptive results examining workforce change by MUA for these cadres was reported.

The MSA level nurse workforce change and the geographically weighted MUA scores were analyzed using a linear regression model to examine the five-year predicted overall change of nurse professions by MUA score at the level of the MSA. To control for population size, all models utilized change rates per 10,000 population as the dependent variable. The analysis used a partially constrained forward selection approach to variable selection and used BLS MSA-level data wherever available in order to maintain geographic consistency. The partially constrained forward selection approach started with a set of theoretically driven variables entered into the model, followed by the iterative addition of remaining variables based on statistical significance criteria as well as availability. A linear ordinary least squares (OLS) regression approach was used to allow for a more nuanced exploration of the two continuous variables of interest (MUA score and growth rates) rather than limiting either to dichotomous values used in logistic regression.

The analysis only used MSAs with sufficient employment and MUA data, as there were five MSAs (approximately 1.4%) that did not report any nurse numbers in 2017 and, as a result, were excluded from the analysis. The growth in the following cadres per 10,000 population was estimated for the regression models: RNs only, NPs only, and RN and NP combined. All models controlled for the following variables: the total number of nurses per population size of each MSA (2017), the percent of the employed population, and median household income (in $1,000 s) lagged average nurse occupation specific wage change for RNs and population size (in 100,000 s), and the percentage of non-white residents in each MSA. The employment and median household income (in thousands) were included in the model to control for the local economic environment. The nurse specific wage change controlled for any nurse workforce change driven by wages and the percent of non-white residents provided an indicator of racial and ethnic diversity across MSAs, which was relevant for understanding structural inequities that may influence the outcomes.

## Results

Table 1 shows the total number of nurses and total number of nurses by nursing occupation over the years 2017–2022 in MSAs in the United States. The total number of employed RNs (non-advanced practice) grew by 5.3% over the 5-year period 2017–2022 and by 24.5% over the decade 2012–2022, reaching a total of 2,689,320 in 2022. The largest change is seen for NPs, increasing 55.1% over the 5-year period and 169.1% over the decade (2012–2022). The second largest increase for nurse occupations is for nurse midwives, increasing 25.5% over the 5-year period and 88.8% over the 10-year period. Nurse anesthetists increase at a more modest rate from 2017 to 2022, recording a 5.3% increase over the 5-year period.

**Table 1:** Total number of nurses by nursing occupation in MSAs and nurse growth rates (2012–2022). Descriptive statistics for total nurses across occupations from 2012 to 2022, with calculated with 5-year (2017-2022) and 10-year (2012-2022) total nurse growth rates

	**RN (Only)**	**Nurse anesthetist**	**Nurse midwife**	**Nurse practitioner**	**RN + NP**
**2012**	2,159,330	17,370	2,420	83,000	2,242,330
**2013**	2,107,140	18,410	2,360	91,560	2,198,700
**2014**	2,208,420	21,430	2,220	99,320	2,307,740
**2015**	2,395,500	25,200	2,830	117,020	2,512,520
**2016**	2,520,550	25,860	3,100	129,530	2,650,080
**2017**	2,554,860	28,370	3,640	143,960	2,698,820
**2018**	2,057,237	24,210	3,340	151,870	2,209,107
**2019**	2,605,250	22,870	3,510	171,020	2,776,270
**2020**	2,616,020	22,580	3,320	181,060	2,797,080
**2021**	2,691,570	25,690	3,810	202,240	2,893,810
**2022**	2,689,320	29,860	4,570	223,340	2,912,660
**5-year growth**	5.3%	5.3%	25.5%	55.1%	7.9%
**10 -year growth**	24.5%	71.9%	88.8%	169.1%	29.9%

**Fig. 1:**
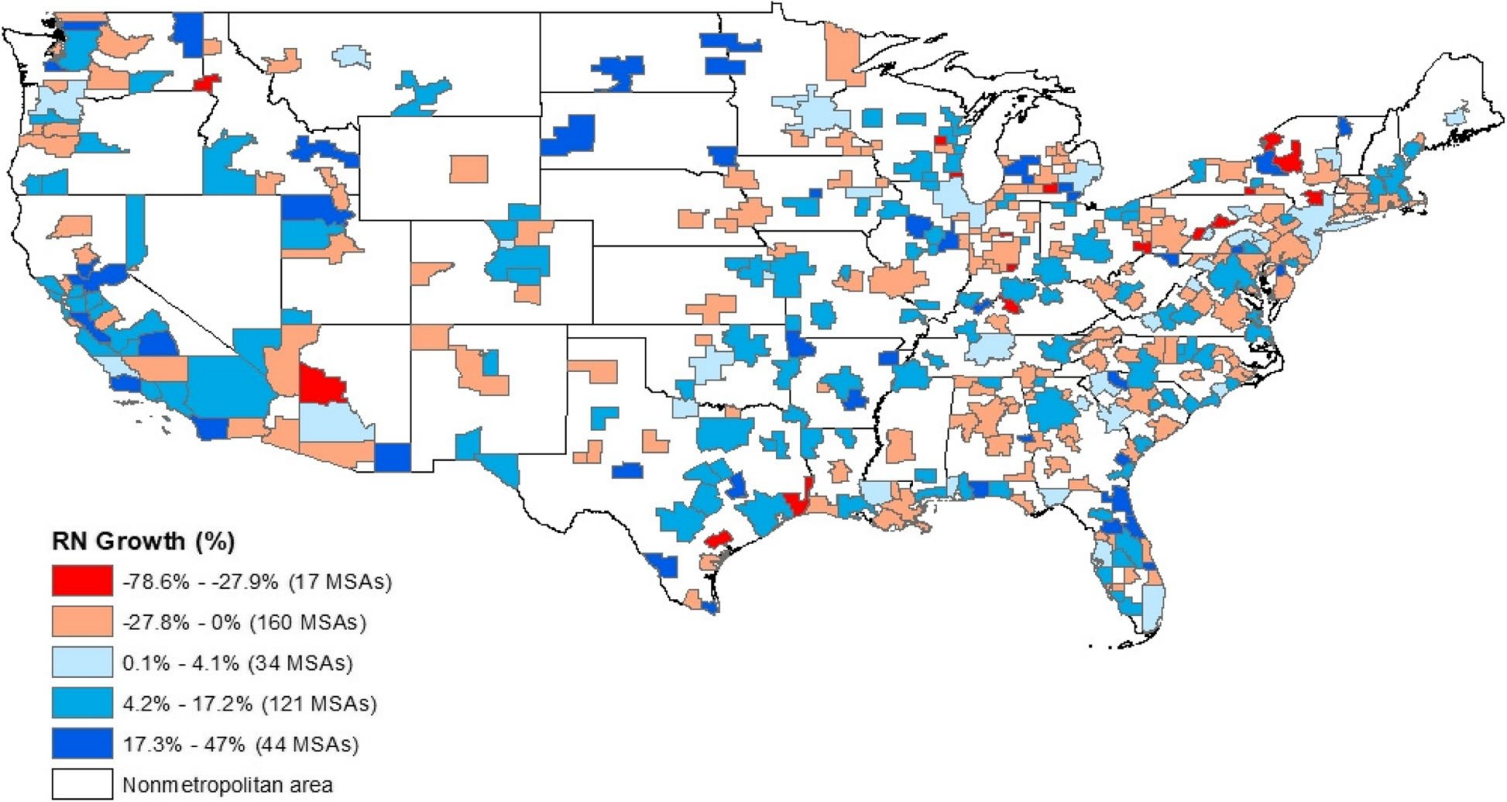
Five-year (2017–2022) Total RN change (%) by MSA

Figure 1 highlights the five-year total RN growth rate by MSA in the US. The map shows that the RN change is focused in a few specific MSAs with most MSAs showing limited or even negative growth in the number of nurses over the period 2017–2022. The five-year total RN change ranges from a 76.8% decrease in southern Texas to over 17.3% increase in North and South Dakota.

Table 2 shows the association (beta coefficients) between MUA score and 5-year change for nurses per 10,000 population for RNs, NPs and a combined model with both RNs and NPs, controlling for employment, income, demographics, population size and nurse wage change. The model shows no significant relationship between MUA score and rates for any of the nurse groups tested, suggesting that despite the large growth in the nursing profession, this growth is not aligned with medical need. The only variables that are correlated with changes in the nurse growth rates are employment, household income, and race (for RN only). The coefficients suggest that for RNs, a one point of change in nurse to population ratio is associated with areas with $1,240 higher household income as well as areas with higher levels of non-white population. NP growth is associated with areas of higher employment but slightly lower levels of income. Even though the effects vary, change in the RN per population ratio is associated with areas with higher employment, higher income, and a higher non-white population. The same analysis is performed for the ten-year period with similar results; however no nurse ratios are significantly associated with MUA scores. Total nurse by occupation breakdowns are shown in Annex Tables A3 and A4.

**Table 2:** Regression results examining the impact of MUA score on 5-year change in nurses per 10,000 population (2017–2022) for RNs, NPs, and RNs and NPs. Regression analysis using BLS OEW data for RN, NP and RN and NP change with MUA score, reporting coefficients and significance level (*p<0.05)

	**RN**	**NP**	**RN and NP**
**MUA score**	0.16	−0.02	0.15
**Employment (%)**	2.58	1.08*	3.6*
**Median Household Income (in $1,000 s)**	1.24*	−0.43*	0.76
**Non-white population (%)**	1.81*	−0.05	1.69*
**Population (in 100,000 s)**	−0.37	−0.01	−0.43
**Average lagged nurse income change**	2.50	−0.36	−0.81
**constant**	257.30*	16.88	−225.85
R-squared	0.06	0.09	0.006
N	346	331	353

**Fig. 2:**
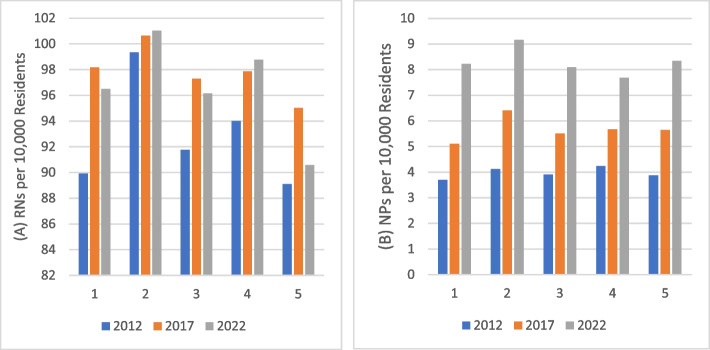
RNs and NPs per 10,000 residents by MUA quintiles for 2012, 2017, and 2022

**Fig. 3:**
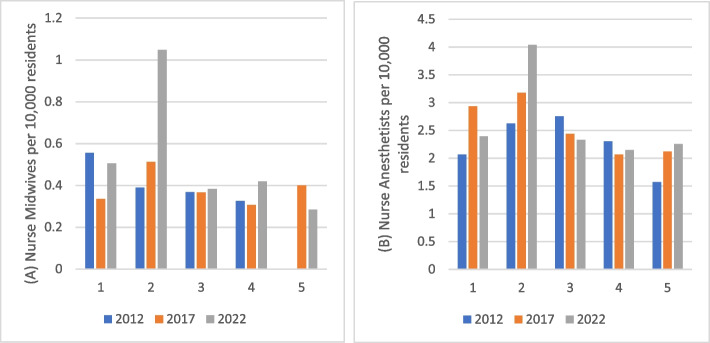
Nurse midwives and nurse anesthetists per 10,000 residents by MUA quintile for 2012, 2017, and 2022

Figure 2 depicts a graph to support the results from Table 2 showing minimal difference in RN and NP change across quintiles. The left-hand side of Fig. 2 supports the results of Table 2, showing a minimal difference in RN density across MUA quintiles. The right-hand side of Fig. 2 shows a slightly higher number of NPs per 10,000 in some of the lower quintiles, especially Quintile 2, but not showing enough variation to produce any significant results. The quintile with the highest number of NPs per 10,000 population (9.2 per 10,000 population) is Quintile 2 in the most recent year, 2022. For both Figs. 2 and 3, the results are graphed by quintile to ease in the visual presentation of the data, showing nurse population ratios in higher and lower MUAs. While the regression analyses did not use quintiles, the patterns in the regression results and the quintiles are similar.

Figure 3 shows the density of nurse anesthetists and nurse midwives per 10,000 population for three years across the different quintiles of geographically weighted MUA scores. The graph shows that there is a higher ratio of nurse anesthetists and nurse midwives in Quintile 2, however, there is little variation across the other quintiles. With the exception of Quintile 2 in 2022, no other quintile has more than one nurse midwife per 10,000 population. While there are insufficient individual MSAs for a reliable regression analysis of both nurse anesthetist or nurse midwife change, the increase in nurse midwives is concentrated in the higher need MUAs while changes in the nurse anesthetist workforce is both inconsistent in distribution and change across MUA levels.

## Discussion

The results of the analysis presented above show that all nursing occupations reviewed have experienced positive change over both five- and ten-year periods. However, the results of the nurse change models reveal that neither NPs nor RNs have been increasing in high medical need areas. These results suggest that existing policies aimed to shift nurse growth to areas of high need have not been effective producing a significant result. These findings suggest possible implications for healthcare access, workforce planning, and payment and policy interventions aimed at addressing healthcare disparities, as discussed below.

Despite the null results in overall findings between nurse change and MUA score, the observed change across the different nursing professions is a positive outcome. The growth in the advanced practice nurse (APRN) workforce mirrors the healthcare industry’s shift to utilizing alternative healthcare professionals, given the physician shortage [37]. The recent growth in the NP profession has been examined in several studies [38, 39]. In comparison, the lower rate of RN growth has been noted by others. Auerbach et al. [16] also find a drop in the number of RNs during the COVID-19 pandemic, and then a rebound after this period. As RNs and NPs play critical roles in primary care delivery, their increased growth, if focused in high medical need areas could help bridge gaps in healthcare access and improve health outcomes for vulnerable populations [40, 41].

A better understanding of nursing workforce change rates, geography, and nurse mobility can be a critical aspect of solving the nursing workforce crisis. For example, examining the location of nursing schools can be an important factor to consider as other research has shown that the location of medical schools and dental schools have been important in retaining doctors and dentists in high need areas [7, 42, 43]. Increasing the interest in the nursing profession among high school graduates can also make a difference, as approximately 52.5% of newly licensed RNs work within forty miles of where they attended high school [44]. Additional analyses should examine these factors as potential policy solutions within the nursing profession.

Another critical component necessary to understanding nurse workforce mobility is the role of loan forgiveness programs. These federal and state level programs vary by state and eligibility criteria, typically offering loan repayment incentives for recently graduated nurses in exchange for working in rural or under-resourced areas to fulfill an unmet need [45, 46]. While prior research has shown loan forgiveness policies to be effective for NP relocation, there are several factors, including having a full practice authority, an individual’s background, and education on working in under-served areas, that impact recruitment and retention in under-served areas generally. Future research should investigate similar and additional factors to understand how RN recruitment and retention in underserved areas can be best addressed by future policies.

Nurse shortages are not limited to the US, and many of the incentive programs mentioned above have been effective internationally [47]. The limitation is that many studies examining efficacy of interventions in underserved areas focus on rural areas, which this study specifically excludes due to limitations in the data. Regardless, some of the strategies may be applicable to metropolitan underserved areas as well. Research in Norway found that placing internships in underserved areas improved worker retention [48]. Programs aimed specifically at creating a pipeline of medical professionals for underserved areas (rural in this case) reduced the shortage in New Zealand [49]. The World Health Organization has a list of recommended incentives that mirror the locating of the training to underserved areas and the recruitment of individuals from those areas as primary goals [50], as well as improving the respect and offering financial incentives to healthcare workers in underserved areas. While there is some evidence that there may be a surplus of healthcare workers in some US areas in the near future, there are and will remain significant areas of shortage [51], further emphasizing the need to tailor policies to better address these geographic inequities. Furthermore, research should explore the long-term impacts of RN and NP recruitment and retention in lower MUAs on health access and outcomes. For example, using longitudinal data to track changes in healthcare workforce distribution over time and disparities in health outcomes or qualitative research examining healthcare provider and patient experiences and perspectives in underserved regions, could also better align strategies with improved retention, access to care and promoting health equity. Improving access to primary care services with a robust nursing workforce can help prevent and manage chronic conditions, reducing emergency room visits, and promoting overall health and well-being [52, 53]. Expanding the scope of RN and NP practice can help reduce physician shortages and improve access to services, particularly when NPs practice to the full extent of their training [40, 54].

Finally, addressing the payment system in the US is another policy option for retaining nurses in the workforce and providing appropriate compensation. Research has shown that with the current prospective payment models, hospitals and other health facilities are not directly reimbursed for high-quality services provided by nurses. Rather, most reimbursable models are linked directly with physician services only [55, 56]. As a result, nurse salaries and labor remain fixed costs for health facilities that need to be minimized [55, 56]. Proposed alternative mechanisms aim to align hospital financial incentives with improving nurses’ work life, by better aligning hospital payments with actual nursing costs and quality [57]. In this way as quality is improved through better nursing care, revenues will also increase. These proposed payment modifications can be utilized to incentivize improving work environment for nurses, better salaries and lower turnover in areas with higher medical need [57].

### Limitations

There are several limitations to the results due to data and methodological or analytical choices made during the research. This analysis is limited to metropolitan areas, and does not include rural areas, and the healthcare workforce dynamics of rural areas differ from their urban counterparts. The findings, while they may reflect the relationship between nurse growth and underserved MUAs, should only be seen within the context of metropolitan MUAs. Additionally, while the MUA score is a generally accepted measurement of medical need, it has many shortcomings, including the explicit exclusion nurses and NPs experts in its determination [58]. There is also the fact that some areas can receive special MUA designation from political leadership without actually qualifying through the empirical measurements, creating the possibility for bias in this analysis. Lastly, due to the limitation of demographic data available at the MUA geographic level, there are few demographic variables that can be included as controls so there are clearly unobserved variables affecting the outcomes. This research has been limited to economic variables. However, it stands to reason that there is a myriad of non-financial motivations that may also drive employment selection, including the location of nursing school, costs of housing, proximity to family, and other quality-of-life measures not fully captured by BLS or MUA data.

## Conclusion

Given the continued healthcare crisis and growing health inequalities in the United States, nurses of all professions are needed in the most vulnerable areas. The results in this paper reinforce the profession's changing dynamics and the need for a deeper understanding of the varying growth trajectories across nursing specializations and geographies. By understanding the factors driving RN and NP growth in lower MUA areas and implementing targeted policy interventions, policies can be tailored towards achieving health equity and ensuring that all individuals have access to the healthcare services they need to thrive.

The lack of significant findings between RN and NP per population ratio changes and medically underserved areas in the regression analyses underscores the complex nature of factors impacting the expansion of nursing occupations. These results emphasize the need for adaptive strategies in the nursing workforce to respond to the evolution of healthcare requirements over time effectively. The findings from this study suggest the need for careful thinking and planning in healthcare policy and education to grow the nurse workforce to effectively to meet the evolving needs of healthcare.

## Supplementary Information

Supplementary Material 1.

## Data Availability

The datasets generated and/or analysed during the current study are available in the Boston College Dataverse Repository, https://dataverse.harvard.edu/dataset.xhtml?persistentId=doi:10.7910/DVN/HQTWXJ.

## Abbreviations

APRN: Advanced Practice Registered Nurse
BLS: Bureau of Labor Statistics
HRSA: Health Resources and Services Administration
HPSA: Health Professional Shortage Area
MSA: Metropolitan statistical area
MUA: Medically Underserved Areas
NP: Nurse practitioner
OEW: Occupational Employment and Wage
RN: Registered nurse
US: United States
