# Supplementary material for: Nurse workforce change and metropolitan medically underserved areas in the United States
Source: BMC Health Serv Res. 2025 Jan 15;25:80. doi: 10.1186/s12913-025-12228-4 (PMC11734408; doi:10.1186/s12913-025-12228-4)
Supplement: Supplementary file 1 — Supplementary Material 1. [file 12913_2025_12228_MOESM1_ESM.docx]

Annex

A1. Geographic distribution of MUAs included in the analysis


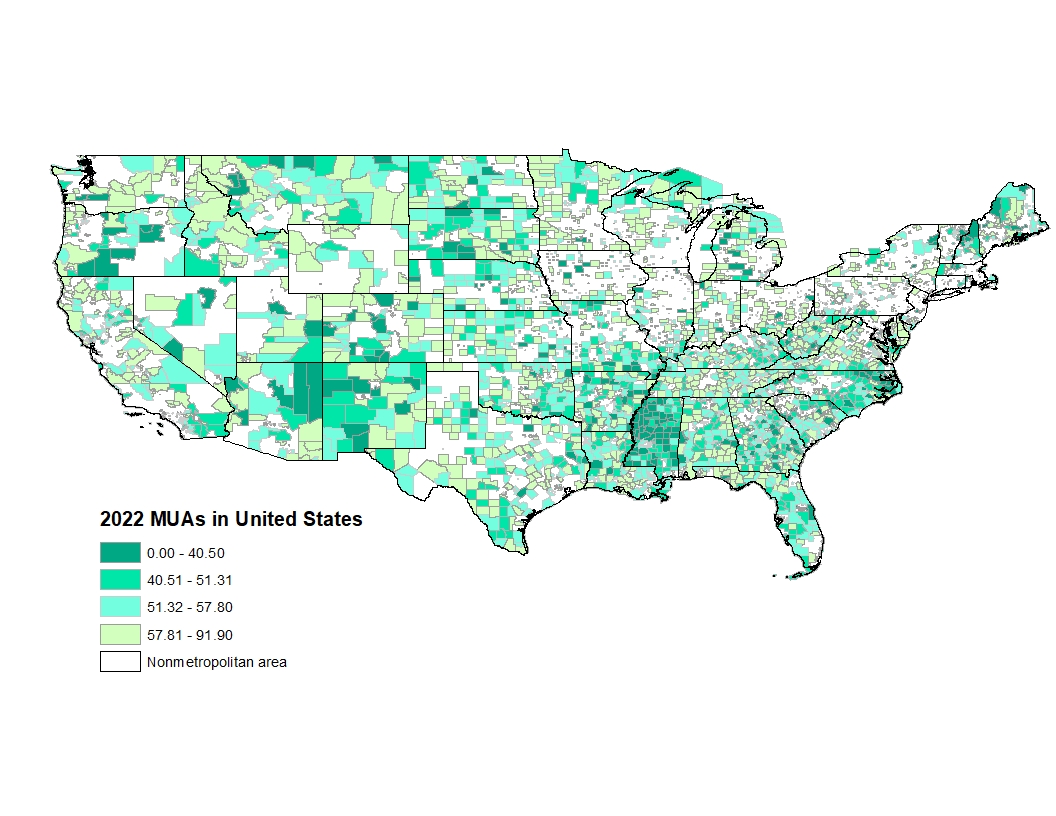


Caption: US map with MUAs color coded by MUA score

A2. Geographically weighted MUA scores within MSA


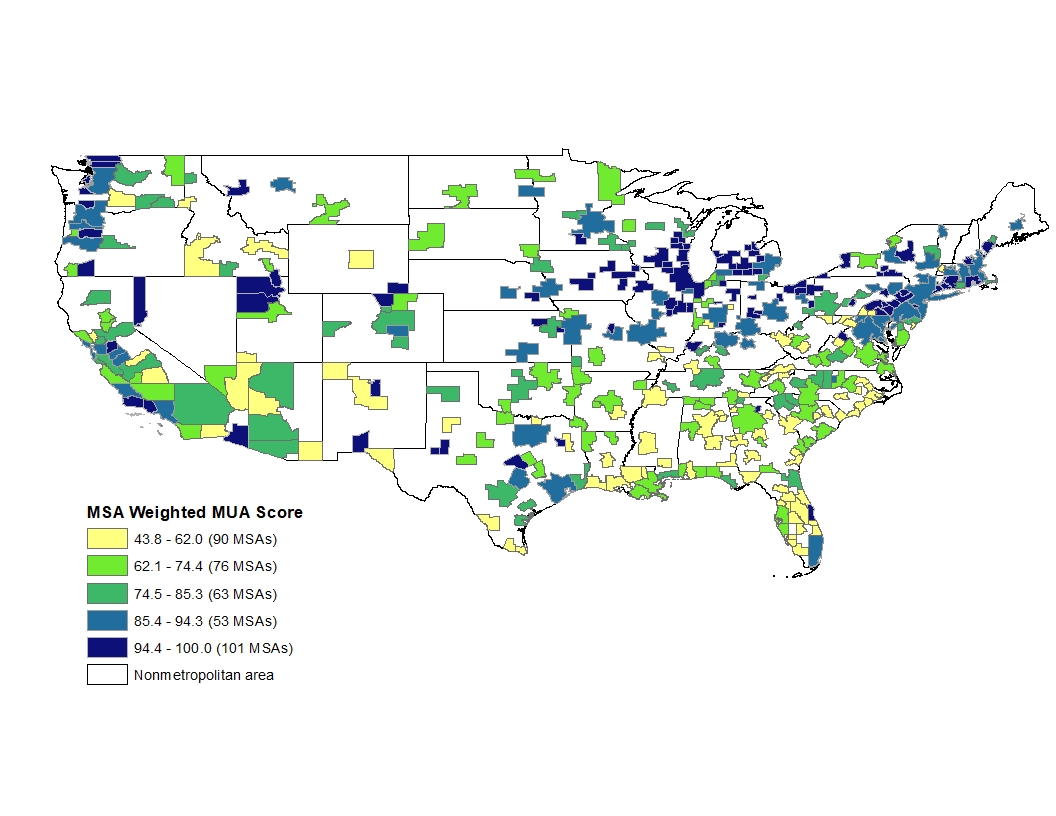


Caption: US map with MSAs color coded by weighted MUA score.

A3. Total number of nurses by nursing occupation (2017-2022)

|  | **RN (Only)** | **Nurse anesthetist** | **Nurse midwife** | **Nurse practitioner** | **APRN** | **All RN** |
| --- | --- | --- | --- | --- | --- | --- |
| **2012** | 2,633,980 | 34,180 | 5,710 | 105,780 | 145,670 | 2,779,650 |
| **2013** | 2,661,890 | 35,430 | 5,460 | 113,370 | 154,260 | 2,816,150 |
| **2014** | 2,687,310 | 36,590 | 5,110 | 122,050 | 163,750 | 2,851,060 |
| **2015** | 2,745,910 | 39,410 | 7,430 | 136,060 | 182,900 | 2,928,810 |
| **2016** | 2,857,180 | 39,860 | 6,270 | 150,230 | 196,360 | 3,053,540 |
| **2017** | 2,906,840 | 42,620 | 6,530 | 166,280 | 215,430 | 3,122,270 |
| **2018** | 2,951,960 | 43,520 | 6,250 | 179,650 | 229,420 | 3,181,380 |
| **2019** | 2,982,280 | 43,570 | 6,930 | 200,600 | 251,100 | 3,233,380 |
| **2020** | 2,986,500 | 41,960 | 7,120 | 211,280 | 260,360 | 3,246,860 |
| **2021** | 3,047,530 | 43,950 | 7,750 | 234,690 | 286,390 | 3,333,920 |
| **2022** | 3,072,700 | 46,540 | 7,950 | 258,230 | 312,720 | 3,385,420 |
|  |  |  |  |  |  |  |

Table shows all the total number of nurses with a consistent growth from 2012 to 2022 for all areas, including metropolitan and nonmetropolitan areas.

| A4. Total number of nurses by nursing occupation in nonmetropolitan areas (2012-2022) | | | | | | |
| --- | --- | --- | --- | --- | --- | --- |
|  | **RN (Only)** | **Nurse anesthetist** | **Nurse midwife** | **Nurse practitioner** | **APRN** | **All RN** |
| **2012** | 317,710 | 1,270 | 30 | 12,540 | 13,840 | 331,550 |
| **2013** | 317,550 | 1,460 | 0 | 13,220 | 14,680 | 332,230 |
| **2014** | 318,380 | 1,390 | 0 | 14,430 | 15,820 | 334,200 |
| **2015** | 283,590 | 970 | 0 | 14,590 | 15,560 | 299,150 |
| **2016** | 295,270 | 1,190 | 0 | 16,350 | 17,540 | 312,810 |
| **2017** | 294,860 | 1,400 | 0 | 17,790 | 19,190 | 314,050 |
| **2018** | 299,800 | 1,050 | 0 | 20,030 | 21,080 | 320,880 |
| **2019** | 296,600 | 1,200 | 0 | 22,650 | 23,850 | 320,450 |
| **2020** | 292,990 | 1,110 | 0 | 24,080 | 25,190 | 318,180 |
| **2021** | 291,950 | 2,350 | 0 | 26,040 | 28,390 | 320,340 |
| **2022** | 290,270 | 2,400 | 30 | 27,870 | 30,300 | 320,570 |

Table shows the total number of nurses in non-metropolitan areas for years 2012 to 2022, showing that there has been an increase of APRNs in nonmetropolitan areas, however there has been a decrease in RNs.
